# Tom Arie, CBE, FRCP, FFPH, FRCPsych (Hon)

**DOI:** 10.1192/bjb.2020.87

**Published:** 2021-02

**Authors:** Claire Hilton

**Professor Emeritus of Health Care of the Elderly, University of Nottingham, UK**

**Figure:**
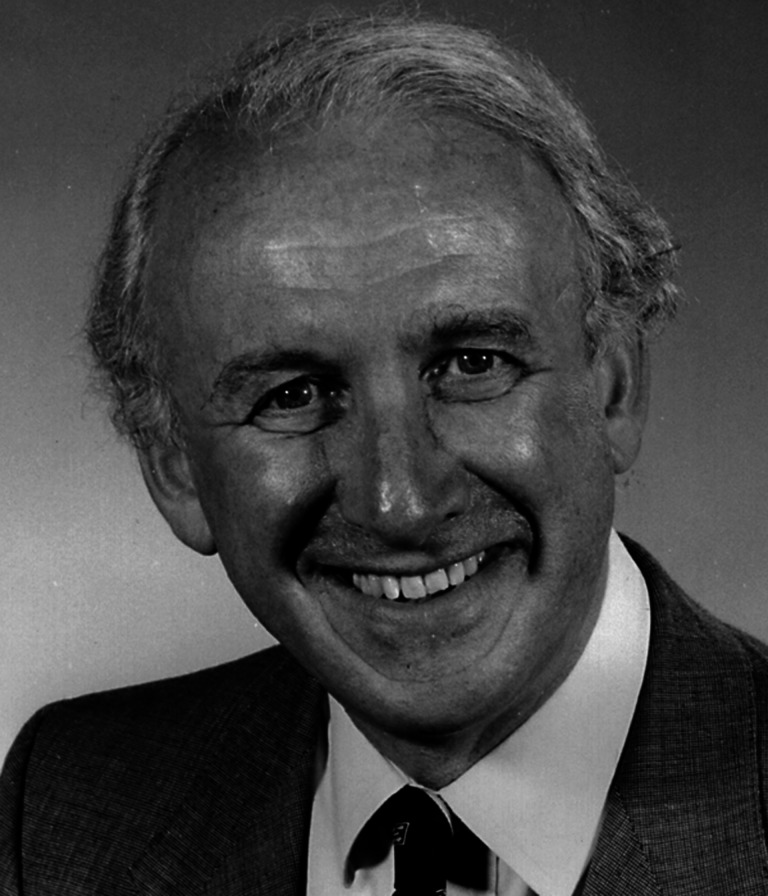

Tom Arie was an old age psychiatrist who steered proactive, treatment-focused mental health services for older people onto the UK policy agenda. This vital step ranks alongside other ‘firsts’ that created the specialty of old age psychiatry: in 1949, Aubrey Lewis established the first dedicated older people's psychiatric assessment ward and appointed Felix Post consultant; in 1955, Martin Roth demonstrated the error of the premise that all mentally unwell older people were ‘senile’; and in 1958, Sam Robinson established the first comprehensive old age psychiatry service in the UK.

Tomas Arje was born into a Jewish family in Prague on 9 August 1933. In 1939, after 5 months living under Nazi occupation, Tom and his parents fled to England, 2 weeks before war broke out.^1^ They settled in Reading, where his parents worked for the BBC, monitoring Nazi broadcasts. In 1952, he went up to Balliol College, Oxford, to read classics. After 2 years, with a first in ‘Honour Moderations’, he followed his growing yearning and swapped to medicine.

Interested in the study of the hospital as a small society, he trained in psychiatry at the Maudsley Hospital, London, and in social medicine at the London (now, Royal London) Hospital. At the Maudsley, working with Felix Post, he found old age psychiatry spellbinding. At the London, Professor Jerry Morris introduced him to social medicine and its leaders. By the time Tom was appointed consultant psychiatrist at Goodmayes Hospital, Essex, in 1969 (‘an unposh place […] Most people thought I had taken leave of my senses!’)^2^ he was personally acquainted with National Health Service and social policy leaders, including Chief Medical Officer Sir George Godber.

Tom and a few other newly appointed consultant psychogeriatricians – a ‘happy band of pilgrims’ as one described them – began to meet. Through Tom's links, they negotiated the content for the Department of Health and Social Security's blueprint for service development, *Services for Mental Illness Related to Old Age* (1972). A year later, and growing in number, the group became the Royal College of Psychiatrists' Group (now Faculty) for the Psychiatry of Old Age. Tom chaired the first meeting – and then supported the election of Felix Post as chair, meanwhile continuing to inspire and nurture behind the scenes.

Away from policies and politics, Tom led an enthusiastic multidisciplinary clinical team characterised by a low hierarchical structure, high morale and positive expectations. Unusually for the 1970s, it included a plethora of part-time women junior doctors, who later became leaders in their own fields. The team pioneered ways to improve the health and well-being of older people, including using domiciliary assessments routinely. They achieved good clinical outcomes and their work highlighted the fallacy of commonplace ageist assumptions. They repeatedly evaluated all aspects of their work in a way that today would be labelled ‘quality improvement’, but for Tom was an instinctive, effective and fruitful way of working.^3^^,^^4^ His creative approach seeped into new teams led by those he inspired.^3^^,^^4^

In 1977, Tom was appointed Professor of Health Care of the Elderly at Nottingham University. There, he modelled and led a joint geriatric–psychiatric team. His department was a magnet and inspiration for politicians and clinicians. In the 1980s, with wide admiration for UK psychogeriatrics and an international lack of trained psychogeriatricians, the British Council, the UK's international cultural relations body, sponsored psychogeriatric courses. These ‘Arie courses’, based on the ‘Arie model’, took place in the UK (Nottingham), Australia, Israel, Korea, Poland, Portugal and elsewhere, creating significant long-term impact. Tom was Vice President of the Royal College of Psychiatrists in 1985–1986. He was made CBE in 1995 for services to medicine. He was awarded a College honorary fellowship in 2001 and the Old Age Faculty lifetime achievement award in 2012. The British Geriatrics Society bestowed their highest honour on him, the Founder's Medal, in 2004.

Tom's goals were humane and idealistic. Some of his ideas were, perhaps, too idealistic, and could not break through bureaucratic, administrative and ideological fashions. He fought for dignity for older people, always sensitive to the moral and medical aspects of their care, not just advocating but also agitating for them**.** Tom's style, and what he enjoyed doing most, was to ‘make grass grow in the desert – enthusing, fostering, encouraging, making things happen, fighting my corner’.^2^ Providing for some of society's most vulnerable people seemed to be an echo from his childhood.

Outside work, Tom was a creator and collector, particularly of friends and paper. He and his wife Eleanor (also a doctor) were always wonderfully hospitable, with their enormous rustic kitchen being the social hub of their home. He read voraciously on many subjects. When a neighbour opened a 24/7 second-hand charity bookshop in her garden shed, he would wander over and browse. He took pleasure in rescuing books from there and the local charity shops that he thought needed love and attention in his hands and on his bookshelves. He kept vast swathes of his personal archives neatly filed, including his diaries; I e-mailed him recently: on what precise date in 1970 did he present a certain report on his work at Goodmayes? The answer flew back a few hours later.

‘I count my blessings every day’, Tom would say. His close-knit family meant the world to him – and he to them – Eleanor and his children Laura, Sophie and Sam, and grandchildren Blake, Zak, Max, Lucy, Eli and Milo, and his wider family. Tom died of cancer at home on 24 May 2020 with his family around him. Now, with his passing, may his memory and his teachings continue to be a blessing to others and inspiration to future generations.
